# The association of physical activity with obesity and NCD outcomes: insights from Kenyan panel data

**DOI:** 10.1038/s41598-026-54585-y

**Published:** 2026-05-30

**Authors:** Cecilia Chemeli Maina, Lukas Kornher

**Affiliations:** 1 Department of Economic and Technological Change, Center for Development Research (ZEF), University of Bonn, Genscherallee 3, 53113 Bonn, Germany; 2 German Institute of Development and Sustainability (IDOS), Tulpenfeld 6, 53113 Bonn, Germany

**Keywords:** Physical activity, Sedentary time, Obesity, Non-communicable diseases, Panel study, Kenya, Risk factors, Obesity

## Abstract

Physical activity (PA) levels in Sub-Saharan Africa are declining due to technological advancements that reduce physical exertion. The aim of this study was to evaluate the longitudinal associations between occupational, leisure, and transport-related PA, as well as sedentary time, on weight and the likelihood of developing a non-communicable disease (NCDs) in Kenya. The study used panel data collected in 2015 and 2022 from 166 participants, selected through a three-stage cluster sampling design. A fixed effects model accounted for unobserved, time-invariant heterogeneity, with results further validated using entropy balancing. Findings indicate declining PA levels, rising sedentary time, and increases in both body mass index (BMI) and NCD incidence. Each additional MET-hour per week of leisure PA is associated with a 0.16% lower BMI, while transport and vigorous occupational PA are associated with 0.052% and 0.025% lower BMI, respectively. A one-unit increase in MET-hours per week of leisure, vigorous occupational, and moderate occupational PA is associated with a 0.53%, 0.15%, and 0.11% lower probability of developing an NCD, respectively. In contrast, sedentary time is positively associated with a 0.18% higher probability of developing an NCD. This study highlights the need for policies promoting PA across all domains, and investments in infrastructure that support active transport and recreational activity to combat obesity and NCDs in Kenya.

**Supplementary Information:**

The online version contains supplementary material available at 10.1038/s41598-026-54585-y.

## Introduction

The increasing prevalence of overweight and obesity in lower and middle income countries (LMICs) warrants concern due to its significant role as a primary risk factor for non-communicable diseases (NCDs) and the resulting financial burden it places on the healthcare system. In Kenya, it is estimated that if the rise in overweight and obesity is halted by 2025, more than 7.4 million new cases of NCDs will be prevented by 2044^1^. Additionally, the projected direct and indirect economic costs of overweight and obesity are expected to increase by 12 to 25 times in low- and middle-income nations by the year 2060^2^. This will impact public healthcare expenditures and lead to constraints on public spending.

Obesity and NCDs are multifaceted, resulting from the interaction of biological and genetic factors, as well as extrinsic contributors such as social, environmental, and behavioral factors^3,4^. Physical activity (PA) is a health-related behavior that is considered a modifiable risk factor for overweight, obesity, and NCDs^5–7^. The World Health Organization (WHO) recommends that adults engage in at least 150 min of moderate PA or 75 min of vigorous PA per week for substantial health benefits^8^. Failure to meet this target would classify an individual as physically inactive and result in poor health outcomes such as hypertension, cardiovascular diseases, various cancers, and type-2 diabetes^9^. Physical inactivity is linked to over 3.2 million deaths each year, with 2.6 million of these deaths occurring in low- and middle-income countries (LMICs)^10^. Additionally, sedentary behavior is a modifiable risk factor that includes any waking activity involving sitting or reclining that is characterized by an energy expenditure of less than 1.5 metabolic equivalents (METs)^11^. It has been identified as a risk factor for all-cause mortality, obesity, and NCDs independent of the level of PA^12–14^.

The main domains of PA include occupational, leisure, transport, and household activities^15^. The PA distribution varies widely by region; in LMICs, occupational, transport, and household activities constitute a majority of PAs, while in high-income countries, leisure-related PA contributes more to total PA^16–18^. The prevalence of labor-intensive industries and a large informal sector results in widespread engagement in physically demanding jobs, such as agriculture, construction, and artisanal occupational, resulting in high levels of occupational-related PA. Transport-related PA contributes a significant portion of PA in sub-Saharan Africa (SSA) because transportation infrastructure may be underdeveloped or unreliable leading to a significant portion of PA being accrued through walking, cycling, or other active forms of transportation^19^. This occurs alongside limited access to leisure sports facilities due to their lack of availability and high costs. It is therefore important to understand the distinctions and contributions of the different types of PAs to better understand how they contribute to overall health outcomes and tailor interventions accordingly.

There have been few empirical studies conducted on the relationship between PA and sedentary behaviors with weight, as well as the probability of an NCD outcome, especially in SSA. Muti et al. (2023) investigated the link between self-reported PA in various domains (occupational, leisure, and transport) and body mass index (BMI). This study analyzed cross-sectional data from 9,388 adult participants in four SSA countries and revealed that meeting PA guidelines of at least 150 min per week was associated with a BMI reduction of 0.82 kg/m² for men and 0.68 kg/m² for women^20^. In other cross-sectional studies within SSA, physical inactivity was also found to be among the predictors of overweight and obesity^21–25^, while sedentary time and low levels of PA were identified as risk factors for NCDs^26–29^. Some studies, on the other hand, suggest that physical activity is not the main driver of rising obesity, which is instead likely driven largely by dietary factors^30–32^.

Previous studies mainly use cross-sectional data which fails to account for biases related to unobserved differences among individuals. This study aims to first analyze the trends in various types of PA over the years utilizing a unique sub-sample panel survey conducted in four counties in Kenya in 2015 and 2022. In this context, examining changes in sedentary behavior over time provides new insights to the literature on PA transitions in SSA. Subsequently, it will contribute to the literature by distinguishing and determining the association between occupational, leisure, and transport-related PA together with sedentary time, on weight and NCD outcomes. We further differentiate between vigorous and moderate occupational-related PA to investigate how varying levels of energy expenditure demands in the work place over time is associated with health outcomes. This study will also take into account the intensity, frequency, and duration of physical activities. The use of panel data will help control for unobserved heterogeneity biases, such as preferences, motivation, and the opportunity cost of time, which may affect both PA and health outcomes. We further provide robustness against observable heterogeneity by utilizing entropy balancing to assess whether participation in PA is associated with health improvements.

This paper addresses two research questions: (1) What is the association of occupational, leisure, and transport-related physical activities on BMI & NCD outcomes? (2) To what extent is an increase in sedentary time associated with higher BMI and an increased probability of NCD outcomes?

## Methods

### Study population

The dataset used in this study is from two waves of surveys conducted in 2015 and 2022, comprising adults aged 18 years and above. The first wave was obtained from the 2015 WHO STEPS survey, which was a nationally representative survey conducted in 2015 to collect information on the determinants and risk factors for NCDs, injuries, and oral health^33^. This survey used a three-stage cluster sampling method. Initially, 200 clusters were selected through an equal probability selection method from the fifth National Sample Surveys and Evaluation Program master sample frame, created by the Kenya National Bureau of Statistics (KNBS). Within each of these clusters, 30 households were chosen to participate in the survey through a systematic sampling method with a random start. Finally, from each of these selected households, one adult was randomly selected to participate in the survey. Each county had 23 clusters, each comprising 30 individuals. The 2015 dataset used during the current study is available in the WHO Central Data Catalog repository under open-access rules: Kenya – STEPS 2015 (WHO Central Data Catalog). The dataset can be accessed at: https://extranet.who.int/ncdsmicrodata/index.php/catalog/643.

The follow-up study was conducted from May to June 2022 in 4 specific counties that were chosen based on their overweight and obesity rates in 2015. The results of the 2015 survey showed that Murang’a, Kiambu, and Nakuru had the highest rates of overweight and obesity (46.5%, 44.6%, and 43.9% respectively) among Kenyan counties^22^. In comparison, the rate of overweight and obesity in Uasin Gishu County was 24.02%. This selection strategy allowed us to capture variation in BMI and related health outcomes across different epidemiological contexts. However, since the sample includes only four counties, caution is warranted in generalizing the findings to all Kenyan counties. The same individuals who had participated in the baseline study conducted in 2015 in the four selected counties were chosen for follow-up interviews. This study was approved by the Ethics Committee of the Center for Development Research (ZEF), University of Bonn (registration code 5b/CM/2022) and granted license to conduct research in Kenya (NACOSTI/P/22/16764). All participants provided written informed consent prior to participation in both survey waves. All procedures were conducted in accordance with relevant institutional guidelines and national regulations governing research involving human subjects.

The final sample consisted of 166 respondents, including 101 women and 65 men, with 116 individuals residing in rural settings and 50 in urban areas. The final analysis included 332 person-year observations. This data was collected in 2022, right after the COVID-19 restrictions were lifted. The participants’ physical activity may have been affected by the recent changes in public health measures. There were also significant attrition rates in urban areas due to the economic impacts of the COVID-19 pandemic. These effects caused individuals who faced job losses in urban regions to return to their rural homes, posing challenges in tracking their phone numbers and whereabouts.

Both surveys collected detailed self-reported information from participants regarding their socio-demographics, the intensity, frequency, and duration of transport, occupational, and leisure-related physical activities, daily sedentary time, health conditions, and dietary behaviors using structured questionnaires. Additionally, survey enumerators took physical measurements of the height and weight of participants for BMI calculations.

### Study variables

#### Outcome variables

The first outcome variable used in the study was BMI, which was obtained by dividing weight by height squared (kg/m^2^). BMI values were transformed to the natural logarithm scale to reduce non-linearity, stabilize variance, and limit the influence of extreme values. This transformation also allows regression coefficients to be interpreted approximately as percentage changes in BMI, which is more intuitive and easier for interpretation. Additionally, BMI scores were categorized into three groups: underweight (< 18.5 kg/m^2^), normal (18.5–24.9 kg/m^2^), and overweight and obese (≥ 25 kg/m^2^) for additional model specifications^34^.

The second outcome variable is a binary NCD outcome variable. It takes the value 1 if the respondent has been diagnosed with either high blood pressure, or diabetes, or has experienced a heart attack or stroke at any point. If none of these conditions apply, it takes the value 0.

#### Physical activity variables

The main explanatory variables in this study were self-reported measures of PA that was collected using the International Physical Activity Questionnaire (IPAQ). Respondents were asked to report whether they engaged in vigorous occupational-related PA (requiring significant physical exertion causing large increases in breathing or heart rate), moderate occupational-related PA (requiring moderate physical effort and resulting in minor increases in breathing or heart rate), and leisure activities such as sports, fitness, and recreational activities and transport-related PA such as walking or cycling for more than 10 min. The occupational-related PA in this study encompassed unpaid household chores, harvesting crops, and fishing or hunting for food. Subsequently, respondents were asked to report their participation (yes/no), frequency (number of days per week), and duration (number of hours per day) in these activities, specifically focusing on activities lasting a minimum of 10 min.

Based on this information, we computed an MET-hours score by taking into account the energy requirements of each activity, measured in metabolic equivalents (METs). The MET is a ratio that compares the metabolic rate of an activity to the resting metabolic rate while quietly sitting, set at 1 kcal·kg⁻¹·h⁻¹^35^. To calculate MET-hours, we multiplied the MET score of an activity by the number of hours and the number of days in a week spent doing that activity. This accounts for both the intensity of the activity and the time spent engaging in it ^36^.

The MET values for each activity used in this study were based on the IPAQ scoring protocol^36^. This protocol contains the average METs for vigorous occupational (8 METs) and moderate occupational (4 METs), cycling (6 METs), walking (3.3 METs), and vigorous and moderate leisure activities (8 and 4 METs). We ultimately obtained values for vigorous occupational, moderate occupational, total transport, and total leisure PA, measured in MET-hours per week. These values represent the energy expenditure from various types of physical activities over a week.

Additionally, the participants provided information about their sedentary behavior. This data included the total amount of time spent in a sedentary position, such as sitting or reclining, during various activities such as occupational, home life, commuting, or socializing with friends. However, it does not include the time spent sleeping. This variable was then converted to daily sedentary time in minutes.

#### Other variables

We included several explanatory variables in our analysis. One of these variables was the “Healthy Eating Behaviors Index,” aimed at accounting for the potential influence of dietary habits on BMI. To construct this index, we performed a principal component analysis (PCA) using standardized data related to dietary behaviors such as frequency of fruit and vegetable consumption, frequency of sugar and salt intake, use of spices, frequency of eating meals outside the home, and consumption of carbonated drinks. The first factor produced was used as the healthy eating behaviors score. Subsequently, the score was divided into quintiles to establish a categorical healthy eating behaviors index, with 1 representing poor dietary behaviors and 5 indicating excellent dietary behaviors.

Other explanatory variables in the analysis were: age and age squared as continuous variables to account for the fact that metabolism changes with age^37^. We categorized age into the following age groups: 18–29, 30–44, 45–59, 60–69, and over 70 years. The asset index, which is an important determinant of PA, was used as a measure of income in this study^38,39^. The index was created based on the yes/no responses on the ownership of household items such as radios, televisions, cars, type of dwelling, and ownership of agricultural land and livestock. These responses were coded as 1 = yes and 0 = no. PCA was then conducted, and the first factor produced was used to generate quintiles with 1 = poor to 5 = richest. Other explanatory variables used were marital status (0 = no/divorced/ widowed and 1 = married), employment status (0 = no, 1 = employed), household size defined as he total number of individuals who usually live in the same household as the respondent, regardless of age or relationship, at the time of the survey and years schooling which is the total number of completed years of formal education by the respondent at the time of the survey, including primary, secondary, and tertiary education, but excluding pre-primary or informal education. Marital status and household size were included as control variables as they are likely to influence both physical activity patterns and BMI outcomes. For example, marital status may be associated with lifestyle behaviors, time allocation, and health-related practices, while household size can reflect resource constraints, labor distribution, and caregiving responsibilities, all of which may affect physical activity and nutritional outcomes.

### Empirical modeling

The main objective of the study was to distinguish and analyze the association between occupational PA, leisure PA, transport PA, and sedentary time on the health outcomes of BMI and NCDs. We first estimate the following panel data regression model for the relationship between PA and BMI:

1 $$\:{H}_{it}={\beta\:}_{0}+{\beta\:}_{2}{\left(WPA\right)}_{it}+\:{\beta\:}_{3}{\left(LPA\right)}_{it}+{\beta\:}_{1}{\left(TPA\right)}_{it}+\:{\beta\:}_{4}{\left(ST\right)}_{it}+\delta\:{X}_{it}+\:\omega\:\tau\:+{\mu\:}_{i}+{\epsilon\:}_{it}$$

In Eq. (1), $\:{H}_{it}$ is the health outcome for individual $\:i$ at time $\:t$, in this case, it is the log of BMI. The main explanatory variables are; $\:{WPA}_{it}$ is the occupational-related PA, $\:{LPA}_{it}$ is the leisure-related PA, $\:{TPA}_{it}$ is the transport-related PA, and $\:{ST}_{it}$ is the sedentary time. $\:{X}_{it}$ is the vector of observable regressors that includes age, age squared, wealth status, household size, marital status, employment status, and the healthy eating behaviors Index. $\:\tau\:$ represents the linear time trend to capture systematic changes in the outcome variable across survey waves that are common to all individuals. With only two waves, this term accounts for differences between the first and second waves not explained by the covariates. $\:{\mu\:}_{i}$ represents the individual-specific effects, and $\:{\epsilon\:}_{it}$ is the idiosyncratic error term. We hypothesize negative and significant coefficient estimates for $\:{\beta\:}_{1}$, $\:{\beta\:}_{2}$, and $\:{\beta\:}_{3}$ to indicate that PA has a negative influence on BMI. We also expect a positive and statistically significant coefficient for $\:{\beta\:}_{4}$, demonstrating that increased sedentary time is associated with a higher BMI.

In the analysis, the models were estimated with fixed effects (FE) estimators. The FE model allows for the main PA regressors to be endogenous and correlated with the time-invariant component of the error $\:{\mu\:}_{i}$, while remaining uncorrelated with the time-varying idiosyncratic error term $\:{\epsilon\:}_{it}$. To accurately estimate the coefficients, we need to control for $\:{\mu\:}_{i}$ through differencing techniques; therefore, we cannot estimate the coefficients of time-invariant variables. The random effects (RE) model assumes that the regressors are exogenous and uncorrelated with $\:{\mu\:}_{i}$. We theorize that PA is not exogenous and can be influenced by unobserved factors such as time preference, motivation, and genetics. These potential influences could lead to biased estimates in the RE model. We expect the FE model to provide consistent estimates. We conducted the Hausman test to determine the presence of unobserved heterogeneity. We found a significant test statistic, suggesting that the FE specification is more appropriate. Additionally, we clustered the standard errors at the individual level. We also estimated heterogeneous fixed-effects models that included interaction terms to capture how the relationship between BMI and total PA varies across sex, age groups, BMI categories, and health status (whether or not the individual has ever been diagnosed with an NCD). To prevent collinearity, we ran separate models for each interaction term.

In the second part of the analysis, we estimated the following probit model of an NCD outcome:

2 $$\:{H}_{i}^{*}={\gamma\:}_{0}+{\gamma\:}_{2}{\left(WPA\right)}_{i}+\:{\gamma\:}_{3}{\left(LPA\right)}_{i}+{\gamma\:}_{1}{\left(TPA\right)}_{i}+\:{\gamma\:}_{4}{\left(ST\right)}_{i}+\rho\:{X}_{i}+{\epsilon\:}_{i}$$

We utilized the latest cross-sectional data from 2022 to assess the influence of PA on an NCD outcome, excluding individuals who reported being diagnosed with an NCD in 2015. In Eq. (2), $\:{H}_{i}^{*}$ is the unobservable latent health stock for individual *i*. Instead, we observe *H*_*i*_ *=1* if *H*_i_^*^ *≥ 1* or otherwise *H*_*i*_ *=0. H*_*i*_ represents whether individual *i* has ever been diagnosed with either high blood pressure, or diabetes, or has had a heart attack or stroke, taking the value of one or zero otherwise. The main explanatory variables are similar to Eq. (1): occupational-related PA $\:\left(WPA\right)$, leisure-related PA $\:\left(LPA\right)$, transport PA $\:\left(TPA\right)$, and sedentary time $\:\left(ST\right)$. $\:{X}_{i}$ represents a vector of individual or household characteristics and $\:{\epsilon\:}_{it}$ is the error term. We clustered the standard errors at the individual level.

### Robustness checks

To further establish whether PA is associated with improvements in BMI status and a lower probability of an NCD outcome, we employ entropy balancing as proposed by Hainmueller (2012). This method uses matching based on entropy balancing to provide robustness against observable heterogeneity. This technique preprocesses data with binary treatments by adjusting the covariate distribution of the control group to resemble that of the treatment group through reweighting to meet specified balancing requirements^40^. Entropy balancing estimates weights from a large set of balance constraints, aiming to minimize the entropy distance metric^41^.

We created binary treatment variables indicating participation in physical activities and matched treated and untreated groups using a maximum entropy reweighting scheme. We selected covariates for reweighting: age, wealth status, household size, marital and employment status, and dietary habits. We set balance constraints (mean, variance, skewness) to align the distributions of these covariates between the treatment and reweighted control groups. Entropy balancing is performed for the PA types (vigorous occupational, moderate occupational, leisure, and transport PA), and the weights are obtained. Finally, regression analysis is conducted, regressing BMI and NCD probability on the treatment indicator, incorporating sampling weights, and controlling for matching variables, to estimate treatment effects.

All the analyses were performed using STATA version 17 SE (Stata Corp LP, TX, USA). The baseline characteristics of the overall population are reported as mean ± standard deviation (SD) for normally distributed continuous variables, and n (%) for categorical variables. Statistical comparisons were conducted using T-Tests and ANOVA, as appropriate. Individual-level fixed-effects models were estimated using the xtreg, fe command. Entropy balancing was implemented using the user-written ebalance program to generate weights that balanced covariates across treatment and control groups prior to estimation. A two-tailed P-value of < 0.05 indicates statistical significance. However, in some tables we also reported results at the 10% level (*p* < 0.10) to indicate marginal significance, given the sample size of our study. This higher threshold was chosen a priori to reduce the risk of Type II errors and to better capture potentially important effects in an exploratory context.

## Results

### Descriptive results

**Fig. 1:**
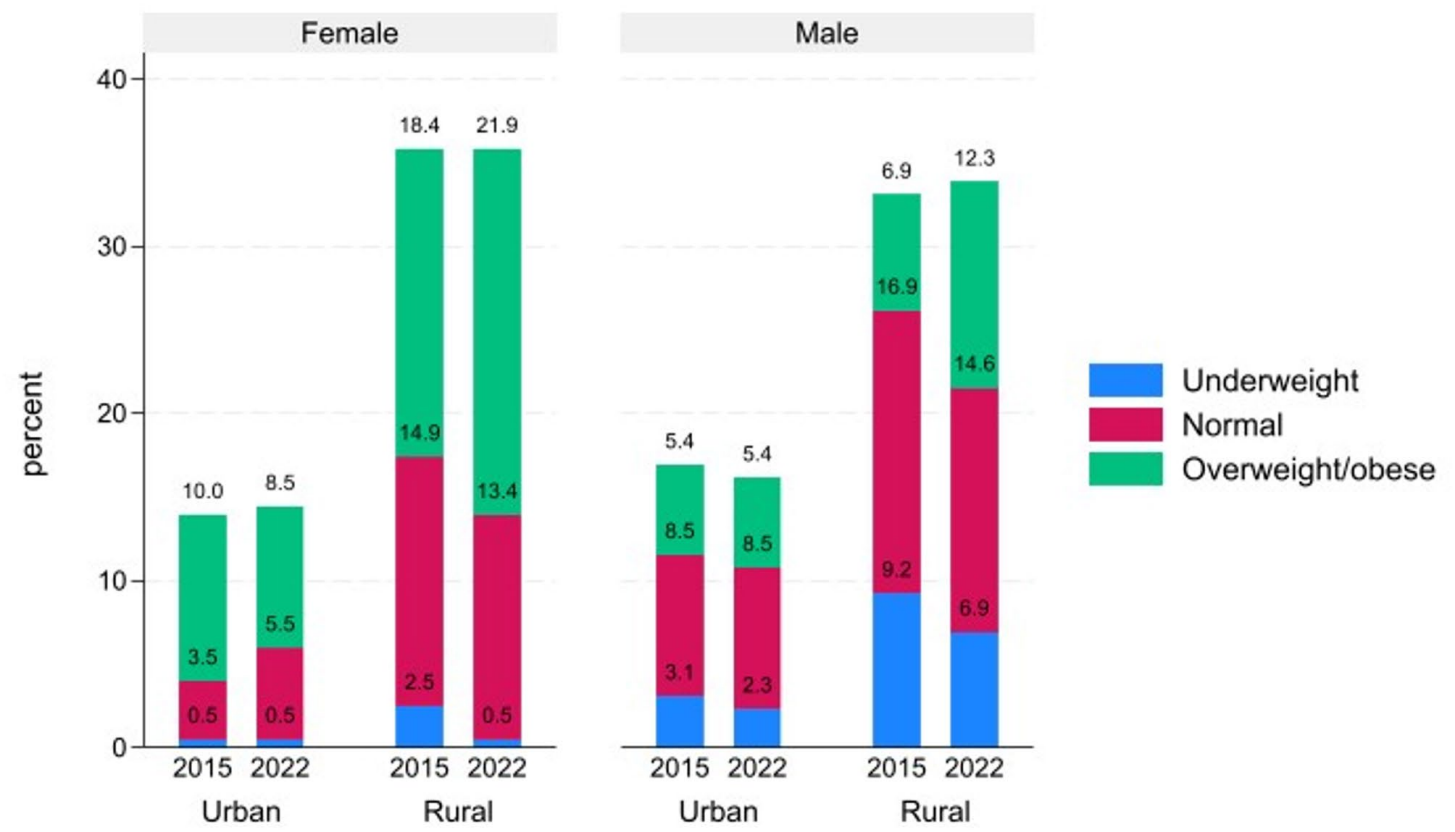
Trends in percentage change in BMI among respondents, 2015–2022.

The summary statistics for the key variables are shown in Table 1. The average BMI for the sample increased from 24.6 kg/m^2^ in 2015 to 25.7 kg/m^2^ in 2022. Both men and women experienced significant increases in the rates of overweight and obesity. Among women, the rate increased from 56.1% to 63.2%, while among men, it increased from 23.4% to 35.1%. Figure 1 illustrates this trend in changes in BMI categories among the respondents over the years. The incidence of NCDs also increased during this period. Among the respondents in the sample, reported diagnoses of high blood pressure increased from 21% to 23%, high blood sugar from 3% to 6%, and reports of a heart attack or stroke rose from 5% to 9%.

**Table 1:** Descriptive statistics for adults.

BMI (Kg/m^2^)	2015 *N*(%)/Mean (SD)	2022 *N*(%)/Mean (SD)	Change (2015–2022)
	24.56 (5.69)	25.73 (5.66)	1.17**
BMI categories	2.31 (0.70)	2.42 (0.64)	0.11
Sex (1 = Male)	0.39 (0.49)	0.39 (0.49)	
Residence (Rural = 1)	0.70 (0.46)	0.70 (0.46)	
Age (years)	42.58 (12.48)	50.05 (12.48)	7.46***
Asset index (5 = Richest)	3.04 (1.13)	2.97 (1.43)	-0.07
Years schooling	8.71 (4.01)	8.88 (4.02)	0.18
Married (1 = Yes)	0.73 (0.45)	0.69 (0.46)	-0.04
Employed (1 = Yes)	0.70 (0.46)	0.81 (0.40)	0.11**
Household size	1.83 (0.91)	2.80 (1.53)	0.97***
Healthy eating behaviors index (5 = excellent)	3.03 (1.46)	2.93 (1.36)	-0.10
Physical activity			
Transportation walk or cycle (1 = Yes)	0.861 (0.347)	0.78 (0.42)	-0.08**
Transportation (MET-hrs/week)	25.04 (37.31)	27.63 (38.63)	2.58
Vigorous occupational (1 = Yes)	0.53 (0.50)	0.45 (0.50)	-0.08
Vigorous occupational (MET-hrs/week)	89.25 (121.40)	56.40 (94.14)	-32.85***
Moderate occupational (1 = Yes)	0.72 (0.45)	0.68 (0.47)	-0.04
Moderate occupational (MET-hrs/week)	47.56 (62.56)	41.21 (61.21)	-6.35
Total occupational (MET-hrs/week)	136.81 (129.61)	97.61 (96.30	-39.20***
Leisure PA (1 = Yes)	0.63 (1.68)	0.31 (1.16)	-0.32**
Leisure PA (MET-hrs/week)	4.46 (14.56)	2.89 (13.10)	-1.56
Sedentary time (Minutes/day)	154.94 (104.20)	176.08 (112.77)	21.14*
Has an NCD	0.25 (0.43)	0.33 (0.47)	0.084*
High blood pressure (1 = Yes)	0.21 (0.41)	0.23 (0.42)	0.02
High blood sugar (1 = Yes)	0.03 (0.17)	0.06 (0.24)	0.03
Had a heart attack or stroke (1 = Yes)	0.05 (0.23)	0.09 (0.29)	0.04
Sample size	166	166	

Standard deviations in parentheses. ***Difference between 2015 and 2022 is significant at 1% level; **Difference between 2015 and 2022 is significant at 5% level; *Difference between 2015 and 2022 is significant at 10% level. Values are presented as means (standard deviations) for continuous variables and proportions (%) for categorical variables. Ordinal indices: higher scores indicate healthier behaviours. Binary variables (Employed, Married, Has an NCD) represent the proportion of participants reporting “Yes.” Statistical tests: t-tests were used for continuous variables; chi-square tests for categorical variables to compare values between 2015 and 2022. Panel note: 2015 and 2022 observations refer to the same sample of participants.

**Fig. 2:**
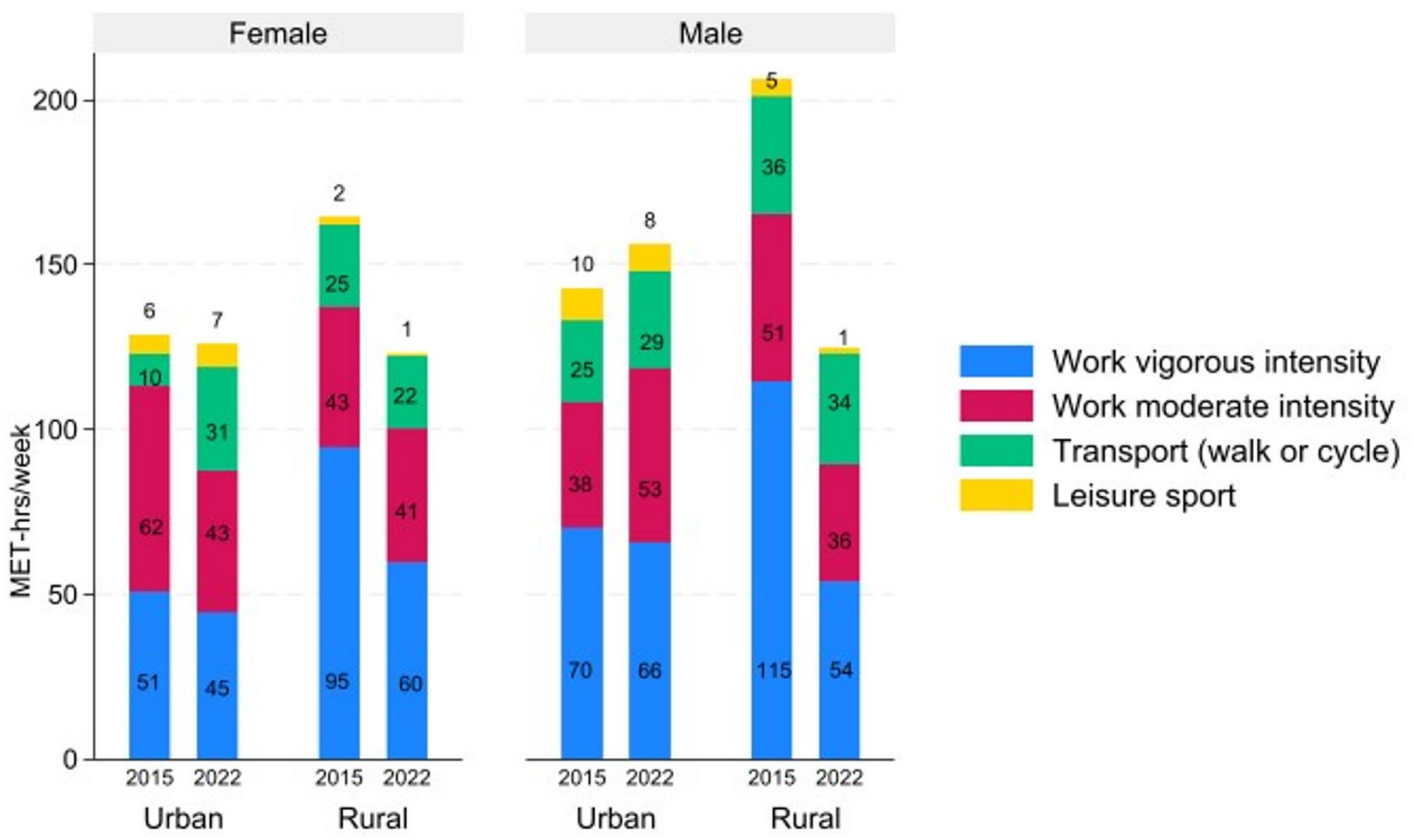
Trends in physical activity levels (MET-hours per week) among respondents, 2015–2022.

In 2022, the main contributors to PA in the sample were occupational-related PA, with 97.6 MET-hours per week, followed by transport-related PA at 27.6 MET-hours per week, and finally leisure-related PA at 2.9 MET-hours per week. Overall, the level of total PA declined in the sample, as shown in Fig. 2. The percentage of individuals who reported engaging in occupational PA decreased in 2022. Occupational-related PA decreased by 39.2 MET-hours per week from 2015 to 2022. Vigorous occupational-related PA declined notably more than moderate occupational PA, dropping by 32.9 MET-hours per week versus 6.4 MET-hours per week. In 2022, fewer individuals (78%) reported walking or cycling for more than 10 min compared to 86% in 2015. Despite this decline, transport-related MET-hours per week slightly increased by 2.6 MET-hours per week during this period. There was also a noticeable decrease in the levels of leisure-related PA within the sample population. The percentage of individuals who reported participating in leisure-related PA decreased from 63% to 31%, and the total number of MET-hours per week decreased from 4.5 to 2.9.

Sedentary time increased from an average of 154.9 min per day to 176.1 min, suggesting that people were spending more time in sedentary activities, which may have contributed to the overall decline in PA levels.

### Regression results

#### Relationship between physical activity and BMI

The results of the panel fixed effects regression models with the log of BMI as the dependent variable are displayed in Table 2. The second column displays the results of BMI regressed against total PA. In column 4, we disentangle PA domains and incorporate additional covariates to control for their confounding effects.

**Table 2:** Relationship between physical activity and body mass index.

BMI kg/m² (log)	(1)		(2)	
	Coef.	SE	Coef.	SE
Total PA (MET-hrs/week)	-0.0033*	0.0018		
Vigorous occupational (MET-hrs/week)			-0.0003***	0.0001
Moderate occupational (MET-hrs/week)			-0.0000	0.0001
Transportation (MET-hrs/week)			-0.0005**	0.0003
Leisure sport (MET-hrs/week)			-0.0016**	0.0008
Sedentary time (minutes/day)			-0.0001	0.0001
Age (years)	0.3752***	0.1375	0.0132**	0.0065
Age²	-0.0045***	0.0014	-0.0002***	0.0001
Asset index (ref: poorest)				
Second	0.9176	0.6146	0.0025	0.0313
Middle	1.1730*	0.6703	0.0207	0.0330
Fourth	1.2932	0.7982	0.0045	0.0426
Richest	2.0747**	0.9481	0.0485	0.0426
Household size	0.1512	0.2038	0.0163**	0.0068
Time trend	1.1265***	0.4245	0.0607**	0.0288
Marital status				
Married (1 = Yes)	0.1165	0.8274	0.1085**	0.0545
Employment status				
Employed (1 = Yes)	0.8305	0.6327	0.0276	0.0278
Healthy eating behaviours index (ref: poor)				
Fair	0.1772	0.6400	-0.0053	0.0328
Good	-0.5183	0.6973	-0.0706**	0.0344
Very good	-0.2583	0.6887	-0.0485	0.0345
Excellent	-0.7157	0.6396	-0.0758**	0.0335
Within R^2^	0.14		0.48	
Number of observations	324		324	

*Significant at 10% level; **Significant at 5% level; ***Significant at 1% level. Coefficients are unstandardised. Robust standard errors are reported.

The results presented in the second column indicate that total PA is negatively and significantly associated with the BMI of adults in Kenya with a coefficient of -0.0029. With the addition of the covariates, vigorous occupational, active transport methods, and leisure-related PA were negatively related to BMI. Since BMI is specified in logarithmic form, the estimated coefficients can be interpreted as approximate percentage changes rather than percentage points. Each additional MET-hour per week of vigorous occupational and leisure-related PA was associated with a 0.025% and 0.16% lower BMI, respectively. Similarly, each additional MET-hour per week of transport PA was associated with a 0.052% lower BMI. Moderate occupational-related PA and sedentary time did not exhibit significant associations with BMI. While these marginal effects appear small, they become more meaningful when considering realistic changes in physical activity levels. For instance, 10–20 additional MET-hours per week, equivalent to several additional hours of moderate-to-vigorous activity, are associated with BMI differences of about 0.25–0.50% for vigorous occupational, 0.52–1.04% for transport activity, and 1.6–3.2% for leisure-related activity. In comparison to other covariates, the magnitude of the physical activity coefficients is similar to or larger than those of several behavioral factors, such as healthy eating categories, but smaller than some demographic variables, such as marital status. This suggests that while the individual marginal effect of physical activity is modest, it remains a meaningful and modifiable determinant of BMI. Relative to baseline BMI levels in the sample, these results represent small but non-negligible changes, particularly when accumulated over time or across the population.

An additional estimation with the dependent variable BMI in 3 categories of underweight, normal and overweight/obese is reported in Table 3. The results indicate statistically significant negative coefficients for vigorous occupational physical activity, leisure sport, and active transport methods. This suggests that these types of physical activities are associated with lower log odds of being in a higher BMI category.

**Table 3:** Effect of physical activity on BMI categories.

BMI (categories)	Pooled OLS		FE		RE	
	Coef.	SE	Coef.	SE	Coef.	SE
Total PA (MET-hrs/week)	-0.0053***	0.0020				
Occupational vigorous (MET-hrs/week)			-0.0106**	0.0047	-0.0098***	0.0025
Occupational moderate (MET-hrs/week)			-0.0049	0.0053	-0.0125***	0.0038
Leisure sport (MET-hrs/week)			-0.0598**	0.0284	-0.0169	0.0146
Transportation (MET-hrs/week)			-0.0473**	0.0224	-0.0144**	0.0074
Sedentary time (min/day)			-0.0046	0.0046	-0.0005	0.0020
Age (years)	-0.0393	0.0706	-0.0175	0.2508	0.2607**	0.1331
Age²	0.0019*	0.0010	0.0010	0.0034	-0.0030**	0.0014
Employed (ref: not employed)	-0.7050	0.7523	-0.3138	0.9748	0.8658*	0.4896
Asset score	0.0393	0.3933	0.4833	0.3816	0.3963**	0.1804
Household size	0.0375	0.4676	0.8904	0.8351	0.2766	0.1744
Married (ref: not married)	1.0724	1.1183	3.2511	2.2401	-0.3076	0.8019
Smoke cigarettes (ref: no smoking)	-1.7438*	1.0128	-3.4605**	1.5280	0.0390	0.9321
Healthy eating (ref: poor)						
Fair	0.3755	0.7836	0.4037	1.1389	1.0899	0.7118
Good	-1.0591	0.6453	0.2272	1.2669	-0.0277	0.6090
Very good	-1.1872	0.7689	-1.8190	1.3221	0.0275	0.6829
Excellent	-1.1946	1.0707	-0.9513	1.3559	-0.3516	0.6441
Has high BP (ref: no)	-0.0517	0.2803	-0.2759	0.3429	-0.3270	0.2290
Has diabetes (ref: no)	0.1576	0.4985	0.0545	0.5245	0.3565	0.2332
Had a heart attack (ref: no)	-0.5266*	0.2886	-1.2919	0.8116	-0.1881	0.1795
Sex × Total PA (interaction)			0.0217*	0.0118	0.0116***	0.0027
Number of observations			178		326	

*Significant at 10% level; **Significant at 5% level; ***Significant at 1% level. Coefficients represent unstandardised effects on BMI category outcomes. The interaction term (Sex × Total PA) captures whether the association between PA and BMI differs by sex. Reference categories: not employed, not married, non-smoker, no NCD diagnosis, and ‘poor’ for the healthy eating behaviours index. Sedentary time is measured in minutes per day. Standard errors are robust (clustered at the household level). Reported sample sizes correspond to the estimation samples for each model.

Table 4 displays the outcomes with interaction terms. The first model examines age groups and total PA, showing that higher PA is associated with lower BMI across younger age categories 18–29, 30–44, and 45–59 (0.052%, 0.024%, and 0.029%, respectively). The second model explored sex and total PA and revealed negative associations between PA and BMI for both men and women (0.041% and 0.016%, respectively). In the third model, interactions between the healthy and NCD groups had negative associations with BMI in both groups. The fourth model assesses the relationship between PA and BMI across BMI categories, indicating lower BMI with higher PA among underweight and normal-weight individuals, while no statistically significant differences are observed for overweight and obese individuals.

**Table 4:** Interactive associations of PA with age, sex, health, and BMI categories on BMI.

	(1) Total PA × Age group	(2) Total PA × Sex	(3) Total PA × NCD outcome	(4) Total PA × BMI category
Total PA × Age group				
18–29	-0.0005*** (0.0002)			
30–44	-0.0002*** (0.0001)			
45–59	-0.0003** (0.0001)			
60–69	0.0000 (0.0002)			
≥70	0.0007 (0.0006)			
Total PA × Sex				
Female		-0.0002* (0.0001)		
Male		-0.0004*** (0.0001)		
Total PA × NCD outcome				
Never diagnosed			-0.0002*** (0.0001)	
Diagnosed			-0.0002** (0.0002)	
Total PA × BMI category				
Underweight				-0.0008*** (0.0001)
Normal				-0.0005*** (0.0001)
Overweight/obese				0.0001 (0.0001)
Observations	324	324	324	324

Robust standard errors are in parentheses. *Significant at 10% level; **Significant at 5% level; ***Significant at 1% level. Total physical activity (Total PA) is measured as MET-hours per week. BMI is measured in kg/m². Reported coefficients represent interaction effects indicating how the association between Total PA and BMI varies across subgroups. Reference categories are male, diagnosed with an NCD, and overweight/obese. All models include the same set of control variables as in Table 2. Standard errors are robust (clustered at the household level).

##### Results of the robustness checks

We initially pre-processed the data using entropy balancing to investigate the link between PA and BMI to derive the estimated treatment effect based on observable factors. We ran four models based on each type of PA. In each model, individuals participating in a particular PA were treated units, while those not engaging in that type of PA served as control units. Covariate distributions of the control group were adjusted using entropy weights to match those of the treatment group. Fixed effects panel regression was then conducted to assess the relationship between the variables, with Table 5 displaying the outcomes. Engaging in vigorous occupational, moderate occupational, and leisure-related PA is associated with lower BMI, with average differences of 3.5%, 3.3%, and 10.4%, respectively. The coefficient for transport-related PA was negative but not statistically significant.

**Table 5:** Association between participation in PA and BMI after entropy balancing.

Treatment	(1) Vigorous occupational	(2) Moderate occupational	(3) Transportation	(4) Leisure sport
Vigorous occupational (MET-hrs/week)	-0.0357** (0.0175)			
Moderate occupational (MET-hrs/week)		-0.0329* (0.0185)		
Transportation (MET-hrs/week)			-0.0146 (0.0262)	
Leisure sport				-0.1040** (0.0420)
Observations	324	324	324	324

Robust standard errors are in parentheses. *Significant at 10% level; **Significant at 5% level; ***Significant at 1% level. The dependent variable (NCD outcome) is a binary indicator equal to 1 if the respondent has been diagnosed with at least one non-communicable disease (e.g., hypertension, diabetes, or heart disease), and 0 otherwise. Reported coefficients are average partial effects (APEs) from probit models. PA variables are measured in MET-hours per week, except leisure sport participation, which is a binary treatment variable. All models include the same set of control variables as in Table 2. Standard errors are robust (clustered at the household level).

#### Association between physical activity on NCD outcomes

In this section, we report the results of a probit regression on the association between PA and the probability of an NCD outcome (diabetes, high blood pressure, and heart attack or stroke). The columns in Table 6 represent the estimated average partial effects of the PA variables. Column 2 shows that total PA is negatively related to the probability of having an NCD. A one-unit increase in MET-hours per week is associated with a 0.08% lower probability of having an NCD. Column 3 represents the coefficients of the PA variables controlling for other covariates. An increase of one unit in MET-hours per week for vigorous occupational, moderate occupational, and leisure-related PA is associated with a 0.15%, 0.11%, and 0.53% lower probability of developing an NCD, respectively. Additionally, sedentary time is associated with a 0.18% higher probability of developing an NCD.

**Table 6:** Association between physical activity and NCD outcome (probit model with entropy balancing, average partial effects).

	(1)	(2)
	Coef. / (SE)	Coef. / (SE)
Total PA (MET-hrs/week)	-0.0022* (0.0003)	
Vigorous occupational (MET-hrs/week)		-0.0015*** (0.0004)
Moderate occupational (MET-hrs/week)		-0.0011* (0.0006)
Leisure sport (MET-hrs/week)		-0.0053*** (0.0019)
Transportation (MET-hrs/week)		0.0002 (0.0007)
Sedentary time (min/day)		0.0018*** (0.0003)
Age (years)	0.0897 (0.0714)	0.0366* (0.0195)
Asset index (ref: poorest)		
Second	0.1291 (0.3738)	-0.0412 (0.0624)
Middle	0.5753 (0.3566)	0.1593** (0.0666)
Fourth	0.5017 (0.3648)	0.2706*** (0.0833)
Richest	0.5066 (0.3729)	0.1135 (0.0754)
Household size	0.1128 (0.0960)	0.0986*** (0.0216)
Marital status (ref: not married)		
Married	-0.2109 (0.2791)	0.2471*** (0.0378)
Employment status (ref: not employed)		
Employed	-0.1114 (0.3170)	0.1670*** (0.0554)
Healthy eating (ref: poor)		
Fair	-0.1700 (0.3724)	-0.0020 (0.0724)
Good	0.0794 (0.4018)	-0.0688 (0.0853)
Very good	0.3342 (0.4080)	-0.0246 (0.0859)
Excellent	0.9378 (0.4355)	0.4724 (0.0756)
Number of observations	146	146

Robust standard errors in parentheses. *Significant at 10% level; **Significant at 5% level; ***Significant at 1% level. The dependent variable (NCD outcome) is a binary indicator equal to 1 if the respondent has been diagnosed with at least one non-communicable disease (e.g., hypertension, diabetes, or heart disease), and 0 otherwise. Reported coefficients are average partial effects (APEs) from probit models estimated after entropy balancing. PA variables are measured in MET-hours per week; sedentary time is measured in minutes per day. Column (1) presents total PA only; Column (2) includes disaggregated PA domains simultaneously. Reference categories are: poorest (asset index), not married, not employed, and ‘poor’ for the healthy eating behaviours index. Standard errors are robust (clustered at the household level).

##### Results of the robustness checks

The results of the probit models after entropy balancing (Table 7) support previous estimates showing a negative relationship between participation in PA and the probability of developing an NCD. We obtain statistically significant negative associations between transport, vigorous occupational, moderate occupational, and leisure-related PA and chronic diseases. Participation in these PAs is associated with a 24.9%, 16.3%, 20.1%, and 20.8% lower probability of having an NCD, respectively.

**Table 7:** Probit estimates of the association between PA and NCD outcomes – Average partial effects after entropy balancing.

	(1)	(2)	(3)	(4)
Vigorous occupational (MET-hrs/week)	-0.164** (0.070)			
Moderate occupational (MET-hrs/week)		-0.201*** (0.069)		
Transportation (MET-hrs/week)			-0.208** (0.087)	
Leisure sport				-0.249*** (0.081)

*Significant at 10% level; **Significant at 5% level; ***Significant at 1% level.

## Discussion

Physical inactivity represents a modifiable risk factor that can be addressed to mitigate the increasing prevalence of NCDs and their major risk factors, such as overweight and obesity. This study builds upon previous research by using panel data that accounts for the unobserved heterogeneity and entropy balancing to adjust for differences in observed covariates between the treatment and control groups, to establish the empirical association between various forms of PA, weight status, and NCD outcomes in Kenya.

The results revealed a decrease in total PA between 2015 and 2022. The main contributors to total PA were occupational and transport-related PA. Additionally, there was an increase of 21.2 min per day in sedentary time, coupled with a corresponding rise in the average BMI of 1.2 kg/m². Concurrently, there was an increase in the prevalence of high blood pressure, high blood sugar, and self-reported incidents of heart attacks or strokes during the same period. This trend aligns with previous research indicating an increased prevalence of physical inactivity globally, with high-income countries recording higher rates than low-income countries^42,43^.

The decrease in PA levels is linked to technological advancements in labor-saving occupational tasks, that can lead to more sedentary occupations or lower energy expenditure at work, reduced household activities, and increased reliance on motorized transportation^43,44^. Overall occupational-related PA has experienced a significant decline, primarily attributed to a sizeable reduction in vigorous occupational activities. This decline can be attributed to the adoption of labor-saving technologies in occupational activities such as farming. In Kenya, the shift towards agricultural mechanization has reduced labor requirements, particularly in tasks such as plowing and weeding^45^. Transportation has also become more motorized; between 2014 and 2022, ownership of motorcycles increased from 7% to 13%, while bicycle ownership decreased from 21% to 16.3%^46,47^. Motorcycles are increasingly preferred due to their affordability, fuel efficiency, and suitability for areas with poor road networks. Additionally, leisure-time activities have shifted towards more passive pursuits like watching television and internet use, as indicated by the increase in household television ownership, which has risen from 35% in 2014 to 50.1% in 2022^46,47^. Broader processes such as urbanization, economic development, shifting social and gender norms, and evolving policy environments may also play important roles in shaping PA patterns, alongside environmental constraints that limit opportunities for active living.

The results indicated negative associations between total PA, vigorous occupational, transport, and leisure-related PA with BMI, with the strongest association observed for leisure-related PA. This is consistent with previous research, Ng et al. (2012) applied dynamic panel estimation techniques on a longitudinal sample from China from 1991 to 1996 and found that an increase of 10 MET-hours per week of occupational and home PA was associated with a weight loss of 0.029 kg^48^. Using panel data, Sarma et al. (2014) found that engaging in leisure-time PA, such as 30 min of walking, was associated with a BMI reduction of 0.14 points in males and 0.20 points in females. Additionally, participation in occupational physical activity was associated with a decrease in BMI of 0.19 kg/m² in males and 0.28 kg/m² in females^49^. Regarding transport-related physical activity, Martin et al. (2015) demonstrated that switching from private motor transport to active travel was associated with a reduction of 0.32 kg/m² in BMI^50^.

Multiple robustness checks support the findings. After employing entropy balancing, it was revealed that participants who engaged in vigorous occupational, moderate occupational, and leisure-related PA exhibited, on average, lower BMIs compared to non-participants. Participating in transport-related PA did not have a significant association with BMI in this model and the strongest association was with leisure PA.

Interaction terms revealed varying relationships between PA and BMI across different groups. Significant negative relationships were observed for younger age groups, both sexes (stronger among men), across health statuses, and within underweight/normal weight categories. This could be because older age groups, especially those above 60 years, showed decreased occupational-related PA, possibly due to factors like injury risk and retirement. The lack of significant negative relationships among the overweight and obese group may be due to physiological differences^51^. Sex differences can be attributed to Men’s higher muscle mass and lower body fat percentage which contributes to greater weight loss associations^52^.

This paper also finds that higher PA is associated with a lower probability of NCD outcomes such as high blood pressure, diabetes, heart attack, or stroke. A unit increase in the number of MET-hours per week in vigorous occupational, moderate occupational, and leisure-related PA is associated with a lower probability of having an NCD with leisure-related PA also exhibiting the strongest association. Conversely, sedentary time is associated with an increase in the probability of developing an NCD. Following entropy balancing, the results indicate significant negative relationships for all types of PA, corresponding to a lower likelihood of developing an NCD. A substantial body of evidence shows that physical activity is associated with reduced risk of NCDs, although the magnitude and significance of these associations can vary across contexts and outcomes. For example, Sarma et al. (2015) found that active leisure PA reduced obesity risk by 5% in Canada, with no significant relationship on diabetes, high blood pressure, or heart disease^53^. Additionally, Humphreys et al. (2011) reported a 2.4% decrease in diabetes risk and a 3.8% decrease in high blood pressure risk with daily leisure PA^38^.

The particularly strong association observed between leisure-time PA and BMI, as well as NCD outcomes, has been shown in other studies^54–56^ and may reflect differences in the intensity, duration, and intentionality of leisure activities compared to occupational or transport-related PA, or alternatively indicate that engagement in leisure-time PA serves as a proxy for higher socioeconomic status or greater health consciousness. Relatedly, the domain-specific effects observed for leisure-time PA on NCD risk suggest the need to disaggregate physical activity by domain rather than relying on composite measures that do not reveal meaningful heterogeneity. Future research should therefore engage more with domain-sensitive frameworks, both to clarify the causal pathways through which leisure-time PA operates and to assess the extent to which observed associations reflect true health benefits versus the influence of socioeconomic and behavioral confounders.

Sedentary time is significantly linked to higher NCD risk, with each additional minute of sitting associated with a 0.18% higher risk. The observed increase in sedentary behavior over time represents an important and novel contribution in this context, where empirical evidence on secular trends in sedentary time remains limited. Studies have demonstrated the adverse link of prolonged sitting on chronic diseases, including cardiovascular disease and diabetes^9,57–61^. Yerramalla et al. (2022) in a study on British participants found a 20% higher risk of cardiovascular disease with a one-hundred-minute increase in sedentary time^58^. Huang et al. (2019) using the 1970 British birth cohort found that prolonged sedentary time was adversely associated with biomarkers of diabetes^59^. In the United States, sedentary behaviors, like excessive television viewing, were associated with increased mortality risk, including cardiovascular and cancer mortality^61^. From a public health perspective, these results highlight the growing importance of addressing sedentary lifestyles for NCD prevention, even in populations where occupational and transport-related physical activity levels have traditionally been high.

### Study limitations

There are several limitations in this study. The use of self-report PA questionnaires can be biased and less accurate compared to objective measures such as accelerometers and pedometers, which provide more precise estimates of the duration and intensity of physical activities. Self-reports are subject to recall bias, social desirability bias, and systematic overestimation, potentially affecting the observed associations. While objective devices are generally more accurate, they can require additional resources and careful implementation, though advances in technology have made them increasingly feasible and affordable for research. The study was also limited in sample size, which might impact the generalizability of the data. The results are also not causal, we only show associations between PA and health outcomes therefore further instrumentation may be necessary to establish causality. We employed entropy balancing to adjust for observable differences between groups, this approach does not fully address potential endogeneity; therefore, the findings should be interpreted as associations rather than causal effects. Another limitation of this study relates to the measurement of physical activity. The analysis is based on earlier guidelines that emphasised activity accumulated in bouts of at least 10 min. However, the 2020 WHO physical activity guidelines no longer impose this requirement, recognizing that all movement contributes to health. As a result, our estimates may underestimate total physical activity, particularly in cases where activity is accumulated in shorter, fragmented bouts. This should be considered when interpreting the magnitude of physical activity levels and their associations with health outcomes. The fixed-effects specification is estimated with only two observations per individual. This restricts within-person variation, which may reduce the precision of the estimates and limit the strength of within-person identification. The study was also conducted in 2022, shortly after COVID-19 restrictions were lifted. This timing may have influenced physical activity in several ways. Gyms and other sports facilities may have remained closed, and work-from-home arrangements could have reduced commuting and occupational-related physical activity, while also increasing sedentary time at home. In some cases, these reductions may have been partially offset by increased home-based or outdoor exercise, resulting in heterogeneous effects. Additionally, disruptions to food supply and healthcare services during the pandemic may have affected dietary habits and the diagnosis and management of BMI and NCDs. There is also the possibility of reverse causality between physical activity, BMI and NCD outcomes. Because the NCD variable reflects lifetime diagnosis, it is possible that individuals who were diagnosed with an NCD prior to the survey year may have subsequently changed their physical activity patterns as a result of their condition, rather than physical activity influencing NCD incidence. Although we attempted to mitigate this concern by restricting the analysis to individuals without an NCD diagnosis in the earlier wave, some degree of reverse causation cannot be fully ruled out. Despite these limitations, the study provides an additional insight into the empirical association between PA and health outcomes in the SSA context.

## Conclusions

The findings in this paper show that participation in all forms of PA is declining over time while the daily sedentary time is increasing. This trend is set to continue with technological innovations reducing the amount of activity required for all activities. This paper aimed to establish the empirical relationship between occupational PA, leisure PA, transport PA, and sedentary time with health outcomes such as overweight, obesity, and NCDs. Findings indicate that PA is significantly associated with health outcomes. Transport-related PA is linked to lower BMI outcomes. Vigorous occupational and leisure-related PA are linked to a reduction in both weight and the probability of an NCD outcome, while moderate occupational-related PA is associated with a lower probability of developing an NCD. Leisure PA shows the strongest association. Sedentary behavior was also significantly linked to the probability of having an NCD.

Promoting PA can help reduce poor health outcomes by lowering BMI and decreasing the risk of NCDs. With rising trends in physical inactivity and sedentary behavior, advocating especially for leisure PA becomes even more crucial. In urban Kenyan contexts, safety concerns, inadequate public infrastructure, and high traffic density remain significant barriers to active transport. Policies that prioritize the development of safe, accessible walking and cycling infrastructure particularly in high-density informal settlements where residents have limited access to private vehicles could meaningfully increase transport-related PA. Similarly, investment in publicly accessible sports facilities and open green spaces must account for the unequal distribution of such resources across urban, peri-urban, and rural areas, where community spaces are often unavailable or unsafe, especially for women and girls.

Given resource constraints common across SSA, community-driven and participatory approaches are likely to be more sustainable and culturally acceptable than top-down interventions. These include community walking groups, locally led recreational programs, and partnerships with schools and faith-based organizations. Whole-school approaches that embed PA into the daily school routine have shown promise in similar low- and middle-income country settings and should be prioritized for younger populations. Healthcare settings also offer an important touchpoint, with brief PA counselling by health workers representing a low-cost, scalable intervention. Mass media and community education campaigns, delivered through radio and mobile platforms given their high reach in Kenya, can help shift public awareness of the health benefits of PA in culturally resonant ways. At the workplace level, encouraging movement breaks, stair use, and where feasible, on-site physical activity spaces can help reduce prolonged sedentary behavior, though such interventions are most relevant to formal employment settings and must be complemented by strategies targeting the large proportion of the population engaged in informal or subsistence work. Taken together, these recommendations call for multi-sectoral action that is responsive to local context, adequately resourced, and co-designed with the communities they aim to serve.

## Supplementary Information

Below is the link to the electronic supplementary material.

Supplementary Material 1

## Data Availability

The 2022 dataset used and/or analyzed during the current study is available from the corresponding author upon request.The 2015 dataset used during and/or analyzed during the current study is available in the WHO Central Data Catalog repository, [Kenya - STEPS 2015 (who.int)](https:/extranet.who.int/ncdsmicrodata/index.php/catalog/247) .
