# Supplementary material for: The association of physical activity with obesity and NCD outcomes: insights from Kenyan panel data
Source: Sci Rep. 2026 May 30;16:16698. doi: 10.1038/s41598-026-54585-y (PMC13221450; doi:10.1038/s41598-026-54585-y)
Supplement: Supplementary file 1 — Supplementary Material 1 [file 41598_2026_54585_MOESM1_ESM.docx]

**Table 1. Regression Coefficients for Physical Activity and BMI Outcomes**

| **Predictor** | **Coefficient** | **Std. Error** |
| --- | --- | --- |
| **Physical Activity Domain** | | |
| Total PA (MET-hrs/week) | −0.0028* | 0.0016 |
| Vigorous work (MET-hrs/week) | −0.0027* | 0.0017 |
| Moderate work (MET-hrs/week) | -0.0000 | 0.0002 |
| Transportation (MET-hrs/week) | −0.0008 | 0.0021 |
| Leisure sport (MET-hrs/week) | −0.0032** | 0.0012 |
| **Sociodemographic Characteristics** | | |
| Age (years) | 0.0083*** | 0.0028 |
| Age² | 0.0003*** | 0.0001 |
| Household size | 0.0064 | 0.0143 |
| **Asset Index (ref: Poorest)** | | |
| Second | −0.0880 | 0.0949 |
| Middle | −0.0659 | 0.0802 |
| Fourth | −0.0102 | 0.1090 |
| Richest | 0.0060 | 0.0939 |
| **Marital Status (ref: Not married)** | | |
| Married | 0.0026 | 0.0823 |
| **Employment Status (ref: Unemployed)** | | |
| Employed | −0.0676 | 0.0694 |
| **Healthy Eating Behaviours Index (ref: Poor)** | | |
| Fair | −0.0066 | 0.0329 |
| Good | −0.0331 | 0.0310 |
| Very good | −0.0079 | 0.0342 |
| Excellent | −0.0399* | 0.0191 |

*Note: * p < 0.05; ** p < 0.01; *** p < 0.001*
